# miR-16 Targets Transcriptional Corepressor SMRT and Modulates NF-kappaB-Regulated Transactivation of Interleukin-8 Gene

**DOI:** 10.1371/journal.pone.0030772

**Published:** 2012-01-24

**Authors:** Rui Zhou, Xiaoqing Li, Guoku Hu, Ai-Yu Gong, Kristen M. Drescher, Xian-Ming Chen

**Affiliations:** 1 Department of Medical Microbiology and Immunology, Creighton University Medical Center, Omaha, Nebraska, United States of America; 2 Tongji Medical School, Institute of Hematology, Union Hospital, Huazhong University of Science and Technology, Wuhan, Hubei, People's Republic of China; University of Crete, Greece

## Abstract

The signaling pathways associated with the Toll-like receptors (TLRs) and nuclear factor-kappaB (NF-κB) are essential to pro-inflammatory cytokine and chemokine expression, as well as initiating innate epithelial immune responses. The TLR/NF-κB signaling pathways must be stringently controlled through an intricate network of positive and negative regulatory elements. MicroRNAs (miRNAs) are non-coding small RNAs that regulate the stability and/or translation of protein-coding mRNAs. Herein we report that miR-16 promotes NF-κB-regulated transactivation of the IL-8 gene by suppression of the silencing mediator for retinoid and thyroid hormone receptor (SMRT). LPS stimulation activated miR-16 gene transcription in human monocytes (U937) and biliary epithelial cells (H69) through MAPK-dependent mechanisms. Transfection of cells with the miR-16 precursor promoted LPS-induced production of IL-8, IL-6, and IL-1α, without a significant effect on their RNA stability. Instead, an increase in NF-κB-regulated transactivation of the IL-8 gene was confirmed in cells following transfection of miR-16 precursor. Importantly, miR-16 targeted the 3′-untranslated region of SMRT and caused translational suppression of SMRT. LPS decreased SMRT expression via upregulation of miR-16. Moreover, functional manipulation of SMRT altered NF-κB-regulated transactivation of LPS-induced IL-8 expression. These data suggest that miR-16 targets SMRT and modulates NF-κB-regulated transactivation of the IL-8 gene.

## Introduction

The innate immune response represents the first line of a host's defense against infectious agents. Cells participating in this response include monocytes/macrophages, neutrophils, and mucosal epithelial cells [1]. These cells express a variety of pathogen pattern recognition receptors, including the Toll-like receptors (TLRs). Upon sensing pathogen-associated microbial patterns, TLRs recruit adaptor proteins and activate downstream signaling cascades, including the nuclear factor-kappaB (NF-κB) and mitogen-activated protein kinase (MAPK) signaling pathways. Activation of these pathways induces production of several proinflammatory cytokines and chemokines that participate in the innate immune response [2]. Prolonged activation of the TLR/NF-κB signaling pathway can have devastating effects on the host and result in tissue damage and chronic inflammatory diseases [3]. In contrast, a delayed or insufficient response of the TLR/NF-κB pathway can result in disseminated disease. Because of the delicate balance that is required to protect the host from both pathogens and immune-mediated damage, cells have developed multiple self-regulatory systems to fine-tune TLR/NF-κB-mediated immune responses [4].

The NF-κB family of transcription factors consists of five members: p50, p52, p65 (RelA), c-Rel, and RelB. In most cells, NF-κB exists in a latent state in the cytoplasm bound to inhibitory IκBs that mask NF-κB's nuclear localization signal. Activation of the NF-κB signaling cascade results in translocation of NF-κB into the nucleus and subsequent expression of pro-inflammatory cytokines and chemokines, as well as antimicrobial effector molecules [5]. Recent studies indicate that NF-κB-mediated transcriptional activity is also regulated by changes in chromatin structure resulting from DNA methylation and histone modifications [6]. Co-activators and co-repressors may also modify core histone amino-terminal tails, resulting in changes in the accessibility of DNA to bind transcription factors, including NF-κB [7], [8]. The silencing mediator for retinoid and thyroid hormone receptor (SMRT, also called NCoR2) is a transcriptional coregulatory protein containing several modulatory functional domains, including multiple autonomous repression domains [9]. SMRT promotes the recruitment of histone deacetylases to the DNA promoters bound by its interacting transcription factors [10], and, thus, serves as a repressive coregulatory factor (co-repressor) for multiple transcription factor pathways [11]. RelA/p65-mediated transcriptional activity is repressed both by SMRT and the nuclear receptor co-repressor complex [11], [12], [13], [14].

MicroRNAs (miRNAs) are short 20–25 nucleotides of RNA molecules that are key post-transcriptional regulators of gene expression [15], [16]. In humans, it is predicted that expression of ∼50% of protein-coding genes is controlled by miRNAs [17]. Recent studies indicate that miRNAs may be critical components of the regulatory networks impacting epithelial immune responses [18], [19]. Transcription of miRNA genes in immune cells can be controlled through pathogen recognition receptors, such as TLRs and the downstream NF-κB and MAPK pathways [19], [20]. Functionally, miRNAs may modulate TLR-mediated immune responses at every step of the innate immune network, including production and release of cytokines and chemokines [19], [20], [21], expression of adhesion and co-stimulatory molecules [19], [22], and feedback regulation of immune responses [19], [20], [23]. We previously described an altered expression profile of miRNAs in human biliary epithelial cells following LPS stimulation or microbial challenge [24], [25]. Of these upregulated miRNAs, miR-16 is predicted to be a critical regulator of TLR-mediated inflammatory responses. Indeed, miR-16 induces rapid degradation of RNAs, which contain AU-rich elements (AREs) in their 3′-untranslated regions (3′UTRs) in HeLa cells [26]. The majority of cytokine and chemokine mRNAs contain AREs within their 3′UTRs, including those cytokines and chemokines associated with the early stages of the immune response, such as tumor necrosis factor-alpha, interleukin-8 (IL-8), and IL-6 [27]. miR-16-mediated degradation of mRNAs requires the miRNA processing components, Dicer, Ago/eiF2C family members, and the ARE binding protein, tristetraprolin, and involves interactions between the sequence UAAUAUU of miR-16 and AREs within the 3′UTRs of targeted mRNAs [26]. In contrast, Li *et al.* recently reported that miR-16 is downregulated during human monocyte-macrophage differentiation, and a decrease in miR-16 levels prevents macrophage hyperactivation by acting to repress the activation of NF-κB target genes [28].

In this study, we investigated the expression of miR-16 in human monocytes and biliary epithelial cells in response to LPS stimulation, and the potential role of miR-16 in controlling LPS-stimulated expression of inflammatory genes. The data we report here demonstrate that LPS stimulation decreased cellular expression of SMRT by upregulating miR-16 in a MAPK-dependent manner. Induction of miR-16 showed no effects on the mRNA stability of selected inflammatory cytokines and chemokines, but promoted NF-κB-regulated transactivation of the IL-8 gene through suppression of SMRT. Together, these data suggest that miR-16 targets SMRT to modulate NF-κB-regulated inflammatory responses in human monocytes and biliary epithelial cells.

## Materials and Methods

### Cell lines, reagents, and antibodies

H69 cells are SV40-transformed human biliary epithelial cells originally derived from a normal liver harvested for transplant. This cell line expresses biliary epithelial cell markers and TLRs consistent with biliary function and was cultured as in previous studies [22], [23]. U937 cells were obtained from ATCC (Manassas, VA) and cultured in RPMI1640 (Invitrogen) medium supplemented with 10% fetal bovine serum (heat inactivated). SC-514 (100 µM, Calbiochem), a potent IKK-2 inhibitor, was used to inhibit NF-κB activation. PD98059 (50 µM, MAPK/MEK inhibitor), SB203580 (10 µM, MAPK/p38 inhibitor), and SP600125 (20 µM, MAPK/JNK inhibitor) were obtained from Calbiochem (San Diego, CA). LPS (*E. coli* strain K12; Invivogen) was used at a final concentration of 1 µg/ml. Actinomycin D was purchased from Fisher Scientific (Pittsburgh, PA). At the utilized concentrations, no cytotoxic effects of any of the chemicals were observed on H69 and U937 cells. Anti-SMRT and anti-p65 were obtained from Bethyl Laboratories (Montgomery, TX) and Millipore (Billerica, MA), respectively.

### Plasmids

The pGL3-basic vector containing IL-8 promoter region was obtained from Dr. A. Bradley (Mayo Clinic, MN, USA). The HA-SMRT was a gift from Dr. Hung-Ying Ka (Case Western Reserve University, OH, USA). Human SMRT siRNAs and control siRNAs were purchased from QIAGEN (Valencia, CA). Complementary 40-mer DNA oligonucleotides containing the putative miR-16 target site within the 3′UTR of human SMRT were synthesized with flanking *Spe*I and *Hin*dIII restriction enzyme digestion sites (Table S1). The annealed oligonucleotides were ligated into the *Spe*I-*Hin*dIII sites of the pMIR-REPORT Luciferase vector (Ambion) for studies examining the potential post-transcriptional luciferase regulation by miR-16 interaction with the SMRT 3′UTR, as we previously reported [22], [23]. As an additional control, a pMIR-REPORT Luciferase construct was generated containing SMRT 3′UTR with two mutations (both CTG to GAC) in the putative seed regions for miR-16.

### Anti-miRs and miRNA precursors

To manipulate cellular abundance of miRNAs, miRNA antisense oligonucleotides (anti-miRs) were used to inhibit miRNA function. Specific miRNA precursors to increase miRNA expression were also used as previously reported [22], [23]. H69 and U937 cells were transfected 0–30 nM of the precursors of miR-16 (Ambion), or anti-miR-16 (Ambion), using the lipofectamine^TM^ 2000 reagent (Invitrogen). Non-specific antisense and precursor (Ambion) miRNAs were used as controls in these studies.

### Measurement of cytokine and chemokine proteins

The Beadlyte® Human Multi-Cytokine Flex Kit (Millipore) was used per the manufacturer's protocol to measure selected cytokines and chemokines (IL-8, IL-1α, IL-6, IL-1β, IL12-p40, G-CSF, and RANTES) in H69 and U937 cells stimulated with 1 µg/ml LPS for 12 h with or without transfection with miR-16 precursors. Results were read on the Luminex® 200^TM^ Instrument (Austin, TX).

### Real-time PCR

For real-time PCR analysis of mature miR-16, total RNAs were extracted using the mirVana™ miRNA Isolation Kit (Ambion). An aliquot containing 0.05 µg of total RNA was reverse-transcribed by using the Taqman MicroRNA Reverse Transcription Kit (Applied Biosystems). Comparative real-time PCR was performed in triplicate with the use of the Taqman Universal PCR Master Mix (Applied Biosystems) on the Applied Biosystems 7500 FAST real-time PCR System. Mature miR-16 specific primers and probes were obtained from Applied Biosystems. Normalization was performed using RNU6B primers, and relative expression was calculated using the comparative cycle threshold (Ct) method [24], [25].

For analysis of pri-miRNAs and mRNAs, total RNA was isolated from cells using Trizol (Ambion) per the manufacturer's instructions. RNAs were treated with the DNA-free^TM^ Kit (Ambion) to remove any contaminating DNA. Comparative real-time PCR was performed using the SYBR Green methodology (SYBR Green PCR Master Mix, Applied Biosystems). Specific primers for pri-miRNAs and mRNAs are listed in Table S1. All reactions were run in triplicate. The Ct values were analyzed using the comparative Ct (ΔΔCt) method [24], [25]. The relative levels of target RNAs were determined by normalizing the reaction to the level endogenous reference glyceraldehyde-3-phosphate dehydrogenase (GAPDH) in each sample and expressed as the relative amount of target to the control (non-treated cells).

### Northern blot

Total RNAs harvested with Trizol reagent were analyzed on a 15% Tris/Borate/EDTA (90 mM Tris/64.6 mM boric acid/2.5 mM EDTA, pH 8.3)-urea gel (Invitrogen) and transferred to a Nytran nylon transfer membrane (Ambion). LNA DIG-probes for miR-16 (Exiqon, Vedbaek, Denmark) were hybridized using UltraHyb reagents (Ambion) according to the manufacturer's instructions, with snRNA RNU6B blotted as a control.

### Measurement of RNA stability

H69 cells were transfected with miR-16 precursors (30 nM) or anti-miR-16 (30 nM) for 24 h, then treated with LPS (1 µg/ml) for 2 h. Transcription was stopped using actinomycin D (10 µg/ml), and RNAs were prepared either immediately or at 0.5, 1, and 2 h post-actinomycin D treatment. Real-time RT-PCR was then performed on the resultant RNA. Each sample was run in triplicate. The relative abundance of mRNAs of IL-8, IL-1α, and IL-6 was calculated using the ΔΔCt method and normalized to GAPDH. The amount of mRNA at 0h following actinomycin D treatment was arbitrarily set to 1. Curve fittings of the resultant data were performed using Microsoft Excel.

### Binding of miR-16 to SMRT 3′UTR

A PCR-based approach previously established to detect direct binding of a miRNA molecule to its targeted mRNA 3′UTR was used [29]. Briefly, total RNA was collected using Trizol (Invitrogen). Reverse transcription was primed with DNA oligonucleotides corresponding to miR-16 sequence (5′-TAGCAGCACGTAAATATTGGCG-3′). Oligonucleotides to miR-143-3p (5′-TGAGATGAAGCACTGTAGCTC-3′) were used as a negative control because no potential binding site for miR-143-3p was identified within SMRT 3′UTR based on complementarity analysis. After reverse transcription, all samples were treated with RNase H at 37°C for 1 h and then amplified with the SMRT gene-specific primers (Table S1). PCR products were resolved in 1.5% agarose gel and stained with ethidium bromide, as previously reported [29].

### Luciferase assay

Cells were transfected with each reporter construct, as well as anti-miR-16 or miR-16 precursor (Ambion), followed by assessment of luciferase activity 24 h after transfection. Luciferase activity was measured and normalized to the expression of the control β-Gal construct, as previously reported [23]. In addition, the full sequence of the IL-8 promoter was cloned into the pGL3-Basic luciferase vector to transfect cells and monitor NF-κB activation.

## Results

### LPS stimulation increases miR-16 gene transcription in a MAPK-dependent but NF-κB-independent manner

**Figure 1:**
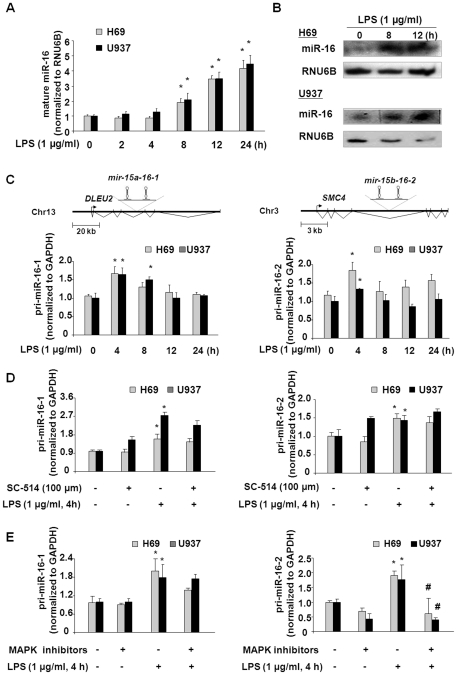
LPS stimulation increases miR-16 gene transcription in a MAPK-dependent but NF-κB-independent manner. (A) Alterations in mature miRNA-16 expression in H69 and U937 cells after exposure to LPS for various periods of time as assessed by real-time PCR. The level of mature miR-16 was obtained by normalizing the reactions to the level of snRNA RNU6B. Data are expressed as the amount of mature miR-16 in LPS-stimulated samples relative to the control non-stimulated samples. Results are averages of three independent experiments. (B) Alterations of miRNA-16 expression in cells after exposure to LPS for 8 h and 12 h, as determined by Northern blot. snRNA RNU6B was used as a control to ensure equal loading between lanes. Representative Northern blots (LPS-stimulated cells vs. non-stimulated control) from three independent experiments are shown. (C) Kinetics of pri-miR-16-1 and pri-miR-16-2 in cells following LPS stimulation. The schematic diagrams show the structure of two miR-16 genes encoding pri-miR-16-1 and pri-miR-16-2, respectively. H69 and U937 cells were exposed to LPS for 4 h to 24 h, and pri-miR-16-1 and pri-miR-16-2 levels were quantified by real-time PCR. (D) and (E) Effects of inhibitors to NF-κB and MAPK pathways on LPS-induced upregulation of pri-miR-16-1 and pri-miR-16-2. The expression levels of pri-miRNA-16-1 and pri-miRNA-16-2 were quantified by real-time PCR in H69 and U937 cells following LPS stimulation for 4 h in the presence or absence of SC-514 or three MAPK signaling pathway inhibitors. The amount of pri-miRNAs in each sample was obtained by normalizing each sample to the level of GAPDH. Data shown are averages of three independent experiments. ∗, p<0.05 *t*-test vs. the non-stimulated cells; ^#^, p<0.05 *t*-test vs. LPS-stimulated cells.

Using microarray analysis, we previously reported altered expression of mature miRNAs in H69 cells following LPS stimulation [24]. Of these specific miRNAs expressed in H69 cells, miR-16 increased following LPS stimulation for 8 h [24]. In the current study, we assessed the kinetics of miR-16 expression in both its mature and primary transcript forms, in H69 and U937 cells following LPS stimulation using real-time PCR. The expression of mature miR-16 did not significantly increase at early time points after LPS exposure in either cell line. Increased mature miR-16 expression was initially detected in cells after exposure to LPS for 8 h (2.0-fold increase), which lasted up to 24 h (4.5-fold increase) as compared to non-LPS treated control cells (Fig. 1A). Increased expression of mature miR-16 following LPS stimulation was further confirmed by Northern blot in both cell lines (Fig. 1B).

Because mature miR-16 is encoded by two separate genes located on chromosomes 13 and 3, which are transcribed to produce pri-miR-16-1 and pri-miR-16-2, respectively (Fig. 1C), we analyzed the kinetics of altered pri-miR-16-1 and pri-miR-16-2 expression in H69 and U937 cells following LPS stimulation. Levels of both pri-miR-16-1 and pri-miR-16-2 showed a time-dependent increase in cells following LPS stimulation (Fig. 1C). Expression of pri-miR-16-1 and pri-miR-16-2 was increased at 4 h after LPS challenge and declined to basal levels by 12 h after LPS stimulation (Fig. 1C).

Similar to the regulation of protein-coding RNA genes, transcription of miRNA genes appears to be regulated by multiple signaling pathways. Activation of the NF-κB and MAPK signaling pathways through TLRs is a common response following LPS stimulation [2]. To determine the role of these signaling pathways in LPS-induced transactivation of miR-16-encoding genes, we measured the levels of pri-miRNA-16-1 and pri-miR-16-2 in H69 and U937 cells in response to LPS in the presence or absence of pharmacological inhibitors to NF-κB or MAPK pathways. Treatment of cells with a specific IKK2 inhibitor, SC-514, inhibits p65-associated transcriptional activation of the NF-κB pathway (Fig. S1) [30], but had no inhibitory effect on the expression of pri-miR-16-1 and pri-miR-16-2 (Fig. 1D). In contrast, cells treated with LPS in the presence of a mix of MAPK inhibitors (50 µM of PD98059, 10 µM of SB203580, and 20 µM of SP600125) showed a significant decrease in pri-miR-16-2, but not pri-miR-16-1, in both cell lines (Fig. 1E). Blockage of nuclear translocation of NF-κB p65 by SC-514, but not the MAPK inhibitors, in cells induced by LPS was confirmed by immunofluorescent microscopy (Fig. S1). To clarify whether TLR4 is required for LPS-induced pri-miR-16 expression, we tested the expression of pri-miR-16-1 and pri-miR-16-2 in H69 cells stably expressing the dominant negative (DN) functionally defective mutant of TLR4 [23]. No significant increase of pri-miR-16-1 and pri-miR-16-2 was detected in cells expressing TLR4-DN following LPS stimulation (Fig. S2). These data suggest that LPS stimulation increases miR-16 gene transcription in H69 and U937 cells through MAPK-dependent but NF-κB-independent mechanisms.

### miR-16 regulates LPS-stimulated expression of IL-8, IL-6, and IL-1α

We then measured the production of TLR-mediated inflammatory cytokines and chemokines at the protein level in H69 and U937 cells following LPS stimulation. Increased production of G-CSF, IL-8, IL-1α, IL-6, and RANTES proteins was detected in H69 cells at 12 h after LPS stimulation. No increase in IL-1β, IL-4, and IL12-p40 protein levels was found (Fig. S3A). LPS stimulation increased protein production of IL-8, IL-1α, IL-6, IL-1β, and IL12-p40 in U937 cells. At the protein level, no changes in G-CSF, RANTES, and IL-4 were detected in LPS-treated U937 cells (Fig. S3B). Of note, increase of IL-1β content in U937 cells following LPS stimulation should cover both pro-IL-1β and the processed mature form of IL-1β because whole cell lysates (not the supernatants) were used for the measurement. In addition, a significant increase in the mRNA levels of IL-8, IL-1α, and IL-6 was also detected in both H69 and U937 cells following LPS stimulation (Fig. S4).

**Figure 2:**
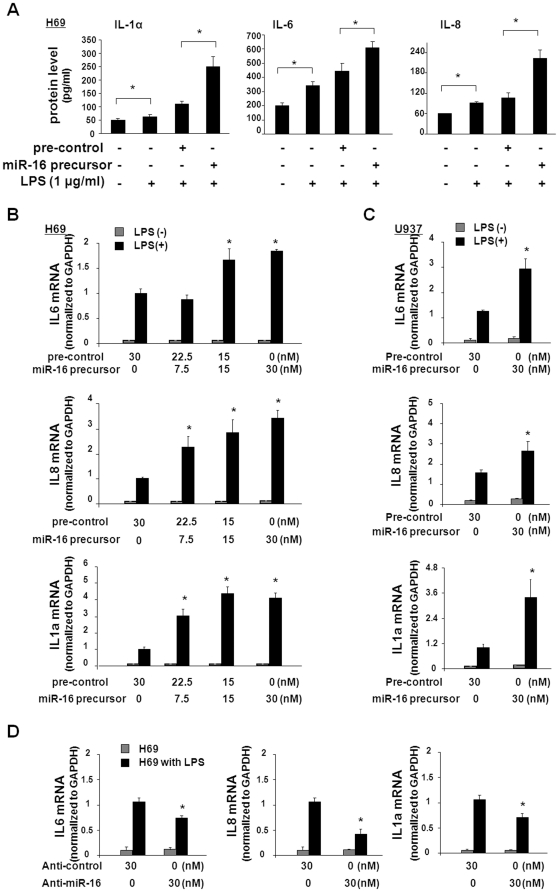
Functional manipulation of miR-16 impacts LPS-stimulated expression of IL-6, IL-8, and IL-1α. (A) Multiplex bead array analysis of IL-6, IL-8, and IL-1α proteins in H69 cells transfected with pre-control or miR-16 precursor for 48 h, followed by exposure to LPS for 12 h. Data shown are averages of three independent experiments. ∗, p<0.05 *t*-test vs. the non-treated control. (B) and (C) Effects of miR-16 precursor on LPS-induced upregulation of mRNAs for IL-8, IL-6, and IL-1α in H69 (B) and U937 (C) cells. mRNA levels of IL-8, IL-6, and IL-1α were measured by real-time PCR in cells transfected with the pre-control or miR-16 precursor for 48 h, followed by exposure to LPS for 6 h. (D) Effects of anti-miR-16 on LPS-induced upregulation of mRNAs for IL-8, IL-6, and IL-1α in H69 cells. Real-time PCR analysis was used to assess IL-8, IL-6, and IL-1α mRNA levels in H69 cells transfected with anti-control or anti-miR-16 for 48 h, followed by exposure to LPS for 6 h. Data are averages of three independent experiments. ∗, p<0.05 *t-*test vs. the controls.

Previous studies demonstrated that miR-16 restricted production of cytokines and chemokines in HeLa cells [26]. To test the impact of miR-16 levels on LPS-regulated expression of inflammatory cytokines and chemokines in H69 cells, we overexpressed miR-16 in H69 cells by transfection of the cells with the miR-16 precursor, and measured the expression of selected cytokines and chemokines following LPS stimulation. Unexpectedly, we detected a significant increase in IL-8, IL-1α, and IL-6 proteins in cells transfected with the miR-16 precursor, compared to cells transfected with the non-specific control precursor (Fig. 2A). Transfection of H69 cells with the miR-16 precursor also significantly increased LPS-stimulated expression of IL-8, IL-1α, and IL-6 at the mRNA levels in a dose-dependent manner (Fig. 2B). A similar increase in IL-8, IL-1α, and IL-6 mRNA levels was evident in U937 cells transfected with the miR-16 precursor (Fig. 2C). In contrast, transfection of H69 cells with anti-miR-16 to inhibit miR-16 function attenuated LPS-induced upregulation of IL-8, IL-1α, and IL-6 mRNAs, compared to cells transfected with the non-specific control anti-miR (Fig. 2D). Taken together, these data demonstrate that miR-16 promotes LPS-induced expression of IL-8, IL-1α, and IL-6 at the mRNA and protein levels in H69 and U937 cells.

**Figure 3:**
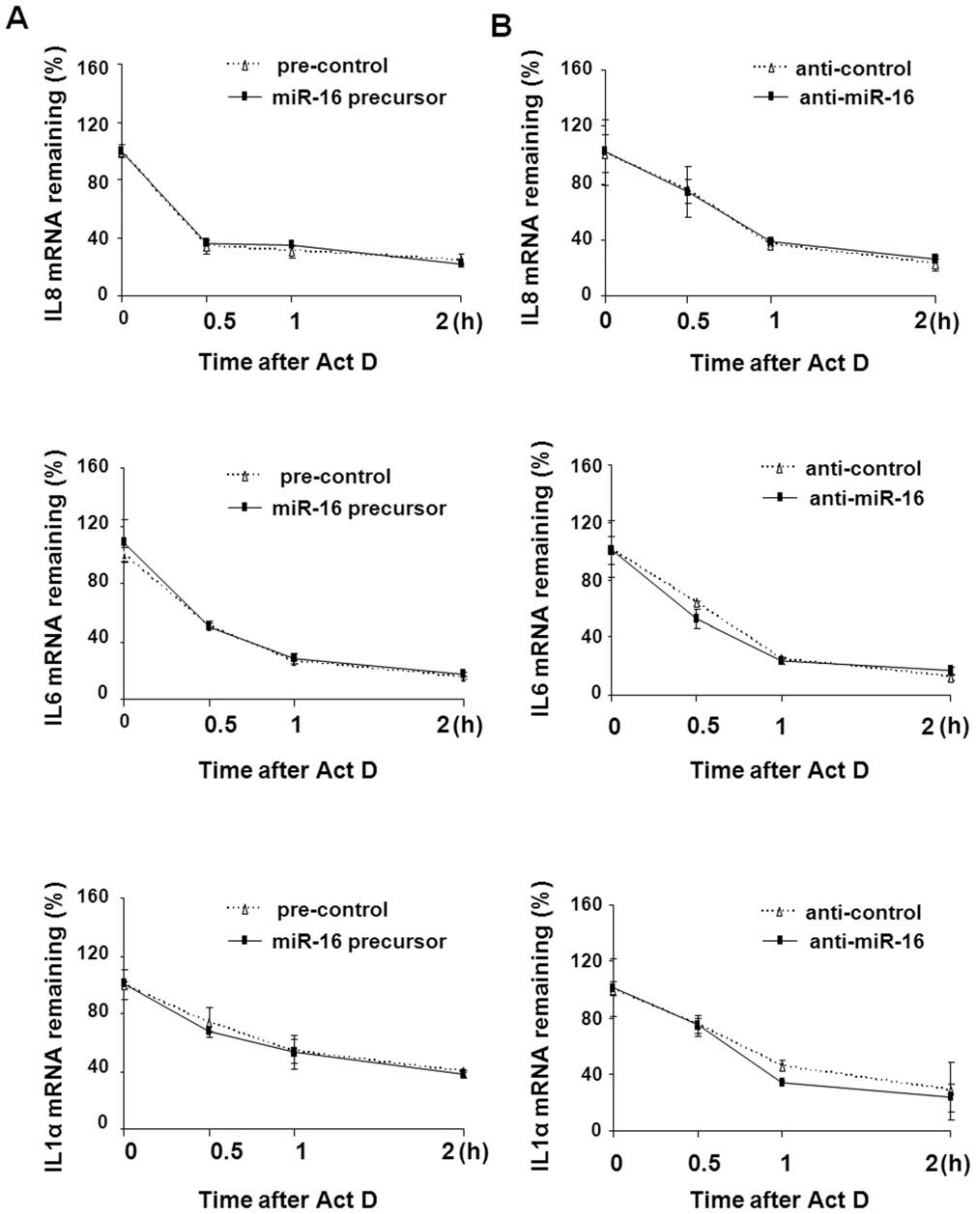
Functional manipulation of miR-16 does not affect the stability of IL-6, IL-8, and IL-1α mRNAs. (A) Effects of the miR-16 precursor on the stability of IL-6, IL-8, and IL-1α mRNAs in cells following LPS stimulation. H69 cells were transfected with pre-control or miR-16 precursor (30 nM) for 24 h, followed by exposure to LPS for 2 h. Act D was then added to the cultures, and cells were collected for real-time PCR analysis. The stability of IL-6, IL-8, and IL-1α mRNAs was calculated and presented as the relative amount of mRNA in cells before Act D treatment. Data represent the average of three independent experiments. (B) Effects of anti-miR-16 on the stability of IL-6, IL-8, and IL-1α mRNAs in cells following LPS stimulation. H69 cells were transfected with anti-control or anti-miR-16 (30 nM) for 24 h, followed by exposure to LPS for 2 h. Act D was then added and RNA stability was measured as described above.

Using HeLa cells, previous studies reported that miR-16 induced degradation of several mRNAs by binding to the AREs of 3′UTRs of the mRNAs [26]. The majority of cytokine and chemokine mRNAs, including IL-8, IL-6, and IL-1α, contain AREs within their 3′UTRs [27]. To test the potential involvement of miR-16-associated RNA destabilization for these particular cytokines, we transfected H69 cells with the miR-16 precursor or anti-miR-16, then measured the mRNA stability of IL-8, IL-1α, and IL-6 following LPS stimulation. As shown in Fig. 3A, the miR-16 precursor had no influence on the mRNA stability of IL-8, IL-1α, and IL-6 after treatment with LPS. Similarly, anti-miR-16 did not influence IL-8, IL-1α, and IL-6 mRNA stability in LPS-stimulated cells (Fig. 3B).

**Figure 4:**
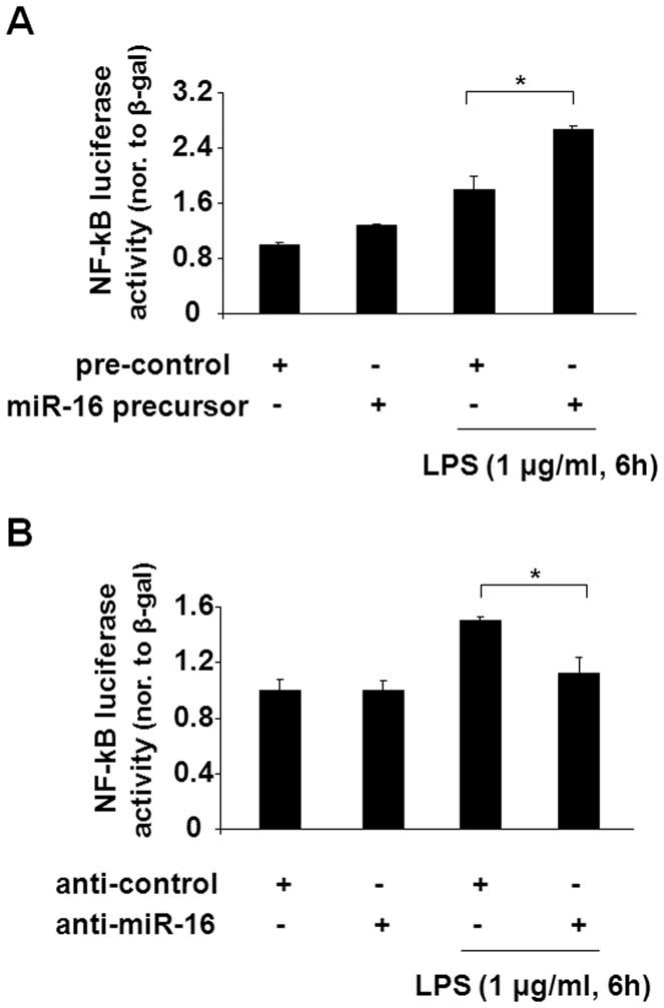
miR-16 enhances NF-κB-regulated transcriptional activity induced by LPS in H69 cells. H69 cells were co-transfected with the NF-κB-driven IL-8 luciferase reporter construct with the miR-16 precursor (A) or anti-miR-16 (B) for 24 h, followed by exposure to LPS for an additional 6 h. Luciferase activity was measured and normalized to the β-gal control. Data shown are the average of three independent experiments. ∗, p<0.05 *t*-test vs. the controls.

Given that IL-8, IL-1α, and IL-6 are all characterized as NF-κB-dependent genes, we then examined the role of miR-16 in NF-κB-regulated transcriptional activity in H69 cells in response to LPS stimulation. NF-κB-regulated transcriptional activity was monitored by using an NF-κB-driven IL-8 luciferase reporter construct as previously reported [23]. H69 cells were co-transfected with the luciferase reporter construct and either the miR-16 precursor or anti-miR-16 for 24 h, followed by exposure to LPS for an additional 6 h. Luciferase activity was measured and normalized to the β-gal control. Transfection of H69 cells with the miR-16 precursor resulted in a significant increase in LPS-induced luciferase activity (Fig. 4A). Complementarily, knockdown of miR-16 using anti-miR-16 caused a decrease in the associated luciferase activity (Fig. 4B). Together, the above data suggest that miR-16 enhances NF-κB-regulated transcriptional activation and promotes production of IL-8, IL-6, and IL-1α in H69 and U937 cells in response to LPS stimulation.

### SMRT is a target for miR-16, and LPS stimulation decreases expression of SMRT via induction of miR-16

To further elucidate the mechanisms underlying the promotion of NF-κB-regulated transcriptional activation in response to LPS by miR-16, we used Targetscan 5.1 (www.targetscan.org) [31] to identify predicted miR-16 targets, focusing on known co-repressors of NF-κB signaling. *In silico* analysis revealed that a miR-16 seed sequence was predicted in one of the highly conserved regions of the 3′UTR of SMRT (Fig. S5). To test whether SMRT is regulated by miR-16, H69 cells were transfected with a specific siRNA to knockdown Drosha, which is required for miRNA maturation [32]. We detected a 1.5-fold increase in SMRT protein levels in Drosha siRNA-treated cells compared to the control siRNA-treated cells (Fig. S6), suggesting miRNA-mediated suppression of SMRT in H69 cells.

**Figure 5:**
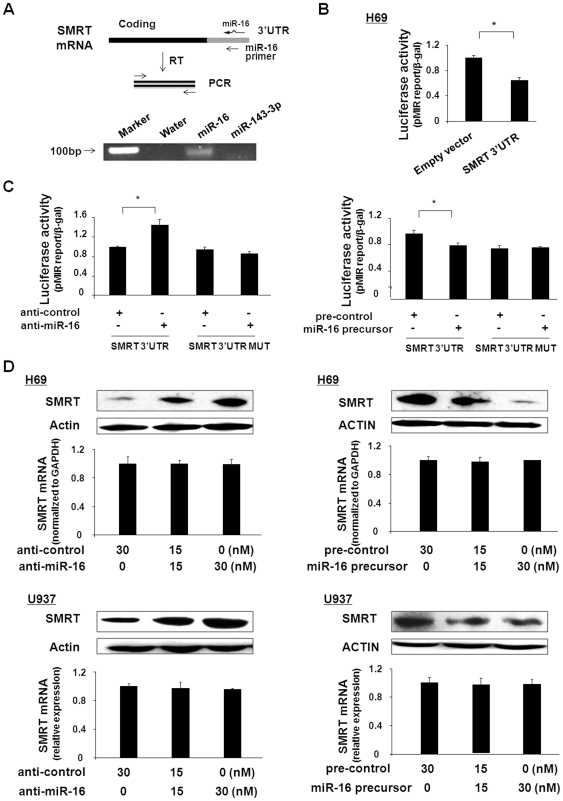
miR-16 targets the SMRT 3′UTR, resulting in translational suppression of SMRT. (A) Binding of SMRT 3′UTR by miR-16 as assessed by a modified RT-PCR approach. The schematic shows the approach using a miR-16 oligonucleotide to prime first-strand synthesis from SMRT mRNA. Following extraction of total RNA from H69 cells, RT was conducted with either a DNA oligonucleotide corresponding to miR-143-3p (negative control) or miR-16, or no primer (negative control), as indicated. Subsequently, PCR was performed with primers specific to SMRT. A 100-bp DNA marker was loaded as a reference. (B) and (C) Targeting of the SMRT 3′UTR by miR-16 results in translational suppression of SMRT in H69 cells. Cells were transfected with the pMIR-REPORT luciferase construct containing the miR-16 binding site in the SMRT 3′UTR, and treated with the anti-miR-16 or miR-16 precursor, followed by luciferase analysis. (D) Manipulation of miR-16 function results in reciprocal alterations in SMRT protein expression in H69 and U937 cells. Cells were treated with various doses of miR-16 precursor or anti-miR-16 for 48 h, followed by Western blotting for the SMRT protein and real-time PCR for SMRT mRNA. Representative Western blots and quantification of SMART mRNA levels from three independent experiments are shown. Blots were standardized using β-actin as a loading control. ∗, p<0.05 *t*-test vs. the controls.

To address whether miR-16 can directly bind to SMRT 3′UTR, we took a PCR-based approach as previously reported [29], using the miR-16 oligonucleotide as a primer in a reverse transcription reaction to examine whether it would prime the SMRT mRNA harvested from H69 cells. The oligonucleotide corresponding to miR-16, but not miR-143-3p, primed first strand synthesis from the SMRT mRNA (Fig. 5A), suggesting that a miR-16 binding site exists in the SMRT 3′UTR. To further test whether miR-16 can bind to the 3′UTR of SMRT and result in translational suppression of SMRT, we generated a pMIR-REPORT luciferase construct containing the SMRT 3′UTR with the putative miR-16 binding site (Fig. S5). In addition, a pMIR-REPORT luciferase construct containing the SMRT 3′UTR with a mutation at the putative miR-16 binding site (CTG to GAC) was generated as the control construct (Fig. S5). H69 cells were transfected with these reporter constructs, and luciferase activity was assessed 24 h after transfection. As shown in Fig. 5B, a significant decrease of luciferase activity was detected in cells transfected with the SMRT 3′UTR construct containing the potential binding site compared with the empty control vector, suggesting endogenous translational repression of the construct with the SMRT 3′UTR. In addition, anti-miR-16 markedly increased SMRT 3′UTR-associated luciferase activity, but did not impact luciferase activity in H69 cells transfected with the mutant SMRT 3′UTR construct (Fig. 5C). In contrast, transfection with the miR-16 precursor significantly decreased luciferase reporter translation (Fig. 5C). The miR-16 precursor had no effect on luciferase activity in cells transfected with the mutant SMRT 3′UTR construct in H69 cells, suggesting that miR-16 may suppress translation of SMRT mRNA by binding to the 3′UTR region of SMRT.

To test whether miRNA-mediated translational repression of SMRT is directly relevant to SMRT protein expression, we treated H69 and U937 cells with anti-miR-16 or miR-16 precursor for 48 h and then measured SMRT protein expression by Western blot. Transfection of H69 and U937 cells with anti-miR-16 caused a dose-dependent increase of SMRT protein content (Fig. 5D). In contrast, a dose-dependent decrease of SMRT protein content was observed in cells treated with the miR-16 precursor (Fig. 5D). Nevertheless, no significant change in SMRT mRNA levels was detected between the control cells and cells treated with miR-16 precursor or anti-miR-16 (Fig. 5D). Coupled with miR-16-mediated translational suppression of SMRT mRNA, our data suggest that miR-16 represses SMRT expression through translational suppression, not RNA degradation.

**Figure 6:**
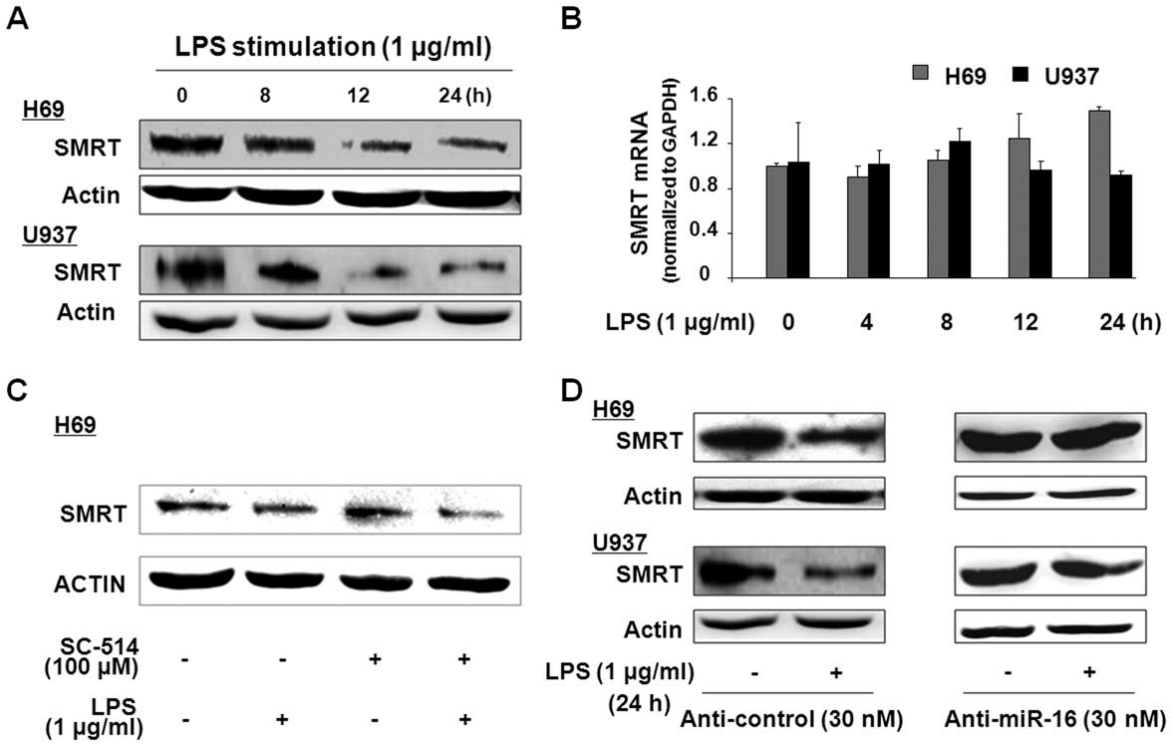
LPS stimulation decreases SMRT expression by upregulating miR-16. (A) and (B) H69 and U937 cells were exposed to LPS for up to 24 h, followed by Western blotting for the SMRT protein and real-time PCR analysis for SMRT mRNA. Downregulation of the SMRT protein (A), but not SMRT mRNA level (B), was detected in both H69 and U937 cells following LPS stimulation. (C) H69 cells were treated with LPS for 24 h in the presence or absence of SC-514 (100 µM), followed by Western blot for SMRT protein. SC-514 showed no significant effects on LPS*-*induced decrease of SMRT. (D) Effects of anti-miR-16 on LPS-induced downregulation of the SMRT protein in H69 and U937 cells. Cells were transfected with anti-control (30 nM) or anti-miR-16 (30 nM) for 24 h and cultured for an additional 24 h in the presence or absence of LPS (1 µg/ml). Representative blots from at least three independent experiments are shown. β-actin was blotted as a loading control.

We then assessed SMRT expression in H69 and U937 cells following LPS stimulation and its correlation to LPS-induced upregulation of miR-16. No significant alterations in SMRT protein level were detected in cells at early time points after LPS stimulation (from 15 min to 6 h) (Fig. S7). Nevertheless, a significant decrease in SMRT protein levels was detectable in H69 and U937 cells following LPS stimulation at later time points, especially after LPS treatment for 24 h (Fig. 6A). When H69 or U937 cells were exposed to LPS for up to 24 h, no significant changes in SMRT mRNA levels were detected, as assessed by real-time PCR (Fig. 6B). Interestingly, SC-514 did not block LPS*-*induced decrease of SMRT protein content in H69 cells (Fig. 6C). To clarify whether miR-16-mediated SMRT translational repression is involved in downregulating SMRT in cells following LPS stimulation, we transfected H69 and U937 cells with anti-miR-16 for 48 h and then exposed the cells to LPS for 24 h, followed by Western blot using an antibody to SMRT. Anti-miR-16 significantly attenuated the downregulation of LPS-induced SMRT protein in H69 and U937 cells (Fig. 6D). Transfection of cells with the non-specific anti-miR control showed no effects on LPS-induced downregulation of SMRT protein (Fig. 6D). The above data suggest that miR-16 upregulation is required for LPS-induced downregulation of SMRT protein in H69 and U937 cells.

### Functional manipulation of SMRT impacts NF-κB-regulated transcriptional activity induced by LPS

**Figure 7:**
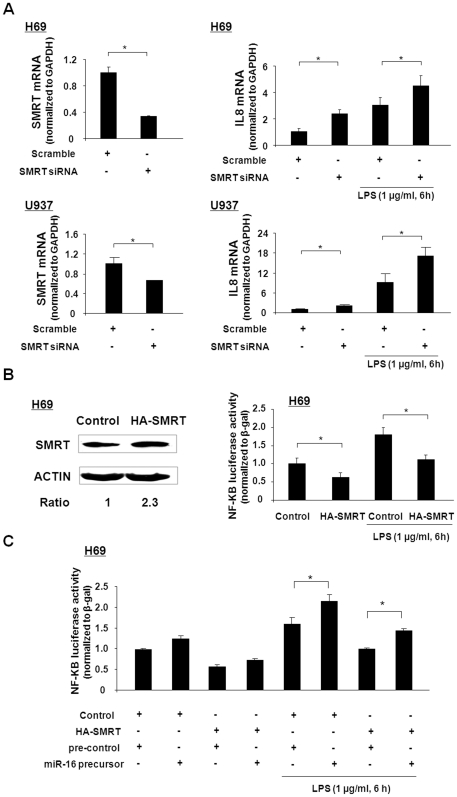
Functional manipulation of SMRT influences NF-κB-regulated transcriptional activity induced by LPS. (A) Effects of SMRT knockdown on LPS-induced IL-8 expression in H69 and U937 cells. Knockdown of SMRT by siRNA was confirmed by real-time PCR analysis. H69 and U937 cells were transfected with the scramble control siRNA or SMRT siRNA for 48 h, followed by an additional 6 h incubation in the presence or absence of LPS (1 µg/ml). Levels of IL-8 mRNA were quantified by real-time PCR. (B) Overexpression of SMRT attenuated LPS-stimulated NF-κB activation in H69 cells. Transfection of H69 cells with the HA-SMRT construct for 24 h increased SMRT expression level by about 2-fold. When cells were co-transfected with full-length SMRT and the NF-κB-driven IL-8 luciferase reporter constructs for 24 h, and then exposed to LPS for 6 h (1 µg/ml), cellular luciferase activity, reflecting NF-κB activation, was monitored. HA-SMRT attenuated LPS-stimulated NF-κB activation in H69 cells. (C) Overrexpression of SMRT attenuated miR-16-mediated NF-κB transcriptional activity induced by LPS. H69 cells were co-transfected with the NF-κB-driven IL-8 luciferase reporter construct with the full-length SMRT plasmid for 24 h, as well as miR-16 precursor, and then exposed to LPS (1 µg/ml) for 6 h. Luciferase activities were measured, and data shown are averages of three independent experiments. ∗, p<0.05 *t*-test vs. the controls.

The SMRT protein has emerged as a negative regulator of the production of cytokines and chemokines [11], [14]. To test whether SMRT regulates NF-κB transcriptional activation and IL-8 production in H69 and U937 cells in response to LPS stimulation, we performed loss-of-function (by siRNA interference) and gain-of-function (by overexpression of SMRT) experiments. Transfection of H69 and U937 cells with SMRT siRNA significantly decreased SMRT expression, resulting in a knockdown of SMRT by 70% and 30%, respectively, over control levels (Fig. 7A). We then measured IL-8 mRNA levels in cells with and without LPS treatment. A significant increase in LPS-induced expression of IL-8 mRNA in cells transfected with the SMRT siRNA, compared to the cells transfected with the control siRNA, was detected (Fig. 7A). Increased IL-8 mRNA levels were also detected in cells transfected with SMRT siRNA but without LPS treatment, compared to cells transfected with the control siRNA (Fig. 7A). In contrast, transfection of H69 cells with the HA-SMRT construct increased the SMRT protein level about 2-fold (with the relative ratio of exogenous versus endogenous SMRT around 2.3), resulting in a significant decrease in LPS-stimulated luciferase activity in cells transfected with the NF-κB-driven IL-8 luciferase reporter construct (Fig. 7B). Similarly, a decrease in NF-κB-regulated luciferase activity was detected in cells overexpressing SMRT without LPS treatment, compared to cells transfected with the control plasmid (Fig. 7B). To test whether miR-16-mediated SMRT translational repression can abolish SMRT-induced repression of NF-κB activity after LPS treatment, H69 cells were co-transfected with the NF-κB-driven IL-8 luciferase reporter construct with or without the miR-16 precursor for 24 h followed by exposure to LPS for 6 h. H69 cells transfected with the miR-16 precursor had increased LPS-stimulated NF-κB transcriptional activity. Overexpression of SMRT inhibited LPS-stimulated NF-κB transcriptional activity (Fig. 7C). Notably, overexpression of SMRT attenuated the effects of the miR-16 precursor on LPS-induced NF-κB transcriptional activation (Fig. 7C). Together, these data suggest that miR-16 regulates LPS-stimulated NF-κB transcriptional activation through repression of SMRT.

## Discussion

In this study, we demonstrated that LPS stimulation increases miR-16 expression in human U937 monocytes and H69 epithelial cells partially through MAPK-mediated transcription of the *mir-15b-16-2* gene. miR-16 targets SMRT 3′UTR and causes translational repression. LPS stimulation decreases SMRT expression by upregulating miR-16. Overexpression of miR-16 promotes NF-κB transcriptional activation induced by LPS through suppression of SMRT, resulting in enhanced production of inflammatory cytokines and chemokines, such as IL-8. These data suggest that miR-16 targets SMRT to modulate TLR/NF-κB-mediated transcription of inflammatory genes, a process that may be involved in the regulation of inflammatory responses during microbial infection of innate immune cells.

miRNA expression may be regulated in a similar manner to that of protein-coding genes, using both transcriptional and post-transcriptional mechanisms [19], [20]. Activation of downstream signaling pathways associated with TLRs may regulate miRNA expression through both mechanisms. In our previous study and those from other groups, a panel of TLR/NF-κB-responsive miRNA genes has been identified in various cell types [19], [20], [24], [25]. TLR-mediated transcription of miRNA genes, such as the *mir-21* gene, is coordinately regulated by multiple downstream signaling pathways, including the MAPK and NF-κB signaling pathways [19], [20], [33]. More recently, it has been reported that the MAPK signaling pathway can promote phosphorylation of the HIV TAR RNA-binding protein, thus enhancing global miRNA maturation by increasing the stability of the miRNA-generating complex [34]. Consistent with previous results [24], we detected upregulation of mature miR-16 in human monocytes and epithelial cells following LPS treatment. We also detected the upregulation of both pri-miR-16-1 and pri-miR-16-2, primary transcripts of mature miR-16 from two miR-16 genes, *mir-15a-16-1* and *mir-15b-16-2,* respectively [24], [26], [35]. Given the correlation of the dynamic kinetics between the mature and primary transcript forms of miR-16, transactivation of the miR-16 genes may account for the upregulation of miR-16 in cells following LPS stimulation. Indeed, NF-κB and MAPK inhibitors had no effects on LPS-stimulated upregulation of pri-miR-16-1. In contrast, MAPK inhibitors, but not an NF-κB inhibitor, attenuated LPS-stimulated upregulation of pri-miR-16-2. In a previous report by Shin *et al.,* upregulation of miR-16 was demonstrated to be dependent on the activation of NF-κB signaling in gastric cells following nicotine treatment [36]. Whether the transactivation of miR-16 genes is differentially regulated by the NF-κB and MAPK signaling pathways in different cell types merits further investigation.

The usual consequence of miRNA and mRNA interactions is decreased protein expression by translational repression and/or mRNA cleavage of the target gene. miR-16 contains a UAAAUAUU sequence that is complementary to the 3′UTR AREs of mRNAs for many inflammatory cytokines and chemokines, such as IL-8, IL-6, and tumor necrosis factor-alpha [26], [27]. Binding of miR-16 to the AREs of these mRNAs promotes ARE-mediated RNA degradation, resulting in destabilization of the targeted mRNAs [26]. In this study, we did not detect a significant impact of miR-16 upregulation on the RNA stability of these mRNAs (IL-6 and IL-8) in human H69 epithelial cells and U937 cells (data not shown) in response to LPS stimulation. Instead, we identified SMRT as a target for miR-16. LPS stimulation of U937 and H69 cells decreases SMRT expression through upregulation of miR-16. Physical interaction between miR-16 and SMRT 3′UTR was demonstrated by RT-PCR using the miR-16 oligonucleotide as a primer in a reverse transcription reaction. Using a luciferase construct containing the sequence in the SMRT 3′UTR predicted to bind to miR-16, we observed translational suppression of SMRT, indicating that miR-16 binding translationally suppresses SMRT. Functional manipulation of miR-16 caused reciprocal alterations in SMRT protein but not SMRT mRNA levels. Anti-miR-16 significantly attenuated LPS-induced downregulation of SMRT in the cells.

SMRT has been demonstrated to function as a regulatory co-repressor of NF-κB transcriptional activity via its association with histone deacetylases recruited to the promoters of NF-κB-regulated genes [11], [13], [14]. Analysis of SMRT-deficient macrophages revealed that SMRT represses subsets of NF-κB-dependent inflammatory genes, including IL-8, IL-6, and IL-1α [11]. Consistent with this observation, we demonstrated that overexpression of SMRT repressed LPS-stimulated NF-κB transcriptional activity in U937 and H69 cells. Accordingly, functional manipulation of miR-16 had a significant impact on LPS-induced production of IL-8, IL-6, and IL-1α. Each miRNA may have multiple mRNA targets in different cell types [15]-[19]. Although miR-16 may have additional targets that could influence LPS-mediated gene expression, our data suggest that miR-16 modulates LPS-induced expression of these genes by targeting SMRT, as SMRT overexpression attenuated the effects of the miR-16 precursor on IL-6, IL-8, and IL-1α expression. Our results are consistent with a recent report by Li *et al,* showing that miR-16 is downregulated during human monocyte-macrophage differentiation, and a decrease in miR-16 prevents macrophage hyperactivation, repressing the activation of NF-κB target genes [28].

From a physiological viewpoint, LPS-induced downregulation of SMRT through MAPK-dependent upregulation of miR-16 and its subsequent effects on NF-κB-mediated transcriptional activity may serve as one component in the network responsible for fine-tuning inflammatory responses in innate immune cells. Because induction of miR-16 and subsequent suppression of SMRT happen at later time points following primary stimulation, such a mechanism may be responsible for resetting chromatin for subsequent rounds of transcription, relevant to cellular inflammatory responses in general. In addition, these data may provide the framework for a new avenue for exploring the cross-talking between the MAPK and NF-κB signaling pathways, both of which are involved in downstream signaling following TLR stimulation. Activation of MAPK signaling has been well-documented to regulate RNA turnover and protein stability of inflammatory effector molecules through post-transcriptional mechanisms [37], [38]. Such activities of MAPK signaling are involved in the regulation of NF-κB-mediated inflammatory responses [27], [38]. MAPK-mediated expression of SMRT through modulation of miR-16 and its subsequent effects on NF-κB-mediated transcriptional activity may provide a new area of exploration for fine-tuning TLR/NF-κB-mediated host reactions in response to microbial challenge.

## Supporting Information

Figure S1 **Effects of inhibitors to NF-κB and MAPK signaling pathways on LPS-induced nuclear translocation.** H69 cells were treated with LPS for 1 h in the presence or absence of inhibitors, followed by immunofluorescent microscopy. p65 was stained in green. Bars  = 5 µm.
(TIF)

Figure S2 **LPS stimulation does not increase expression of pri-miR-16-1 and pri-miR-16-2 in TLR4-DN H69 cells.** The expression of pri-miR-16-1 and pri-miR-16-2 was measured by real-time PCR in H69 cells stably expressing TLR4-DN following LPS stimulation (1 µg/ml) for 4 h. Data shown are averages of three independent experiments.
(TIF)

Figure S3 **LPS stimulation increases expression of inflammatory cytokines and chemokines in H69 and U937 cells.** Multiplex bead array analysis of inflammatory cytokines and chemokines in H69 (A) and U937 (B) cells following LPS stimulation (1 µg/ml) for 12 h. Data shown are averages of three independent experiments. ∗, p<0.05 *t*-test vs. non-stimulated cells.
(TIF)

Figure S4 **LPS stimulation increases mRNA levels of IL-6, IL-8, and IL-1α in H69 and U937 cells.** (A) Time-dependent increase of IL-8 mRNA levels in U937 cells in response to LPS stimulation. (B) and (C) mRNA levels of IL-8, IL-6, and IL-1á were measured by real-time PCR in H69 and U937 cells following LPS stimulation (1 µg/ml) for 6 h. Data shown are averages of three independent experiments. ∗, p<0.05 *t*-test vs. the non-stimulated cells.
(TIF)

Figure S5 **The schematic of SMRT mRNA indicates a potential binding site for miR-16 in the 3′UTR.** The SMRT 3′UTR sequence encoding the potential miR-16 binding site was inserted into the pMIR-REPORT luciferase plasmid. A control plasmid with a mutant 3′UTR sequence was used as a control.
(TIF)

Figure S6 **Drosha silencing induces the upregulation of SMRT expression at the protein level.** H69 cells were transfected with a scramble control siRNA or Drosha siRNA for 48 h, followed by Western blot for SMRT. A representative Western blot from three independent experiments is shown. β-actin was also blotted to ensure equal loading. Densitometric levels of SMRT signals were quantified and expressed as their ratio to β-actin.
(TIF)

Figure S7 **LPS stimulation does not decrease SMRT expression at early time points after LPS treatment.** H69 cells were exposed to LPS for up to 8 h, followed by Western blotting for the SMRT protein. Representative blots from at least three independent experiments are shown. β-actin was blotted as a loading control.
(TIF)

Table S1 **Primers used for PCR and sequence used for construct generating.**
(TIF)
