# Supplementary figures and images for: miR-16 Targets Transcriptional Corepressor SMRT and Modulates NF-kappaB-Regulated Transactivation of Interleukin-8 Gene
Source: PLoS One. 2012 Jan 24;7(1):e30772. doi: 10.1371/journal.pone.0030772 (PMC3265513; doi:10.1371/journal.pone.0030772)

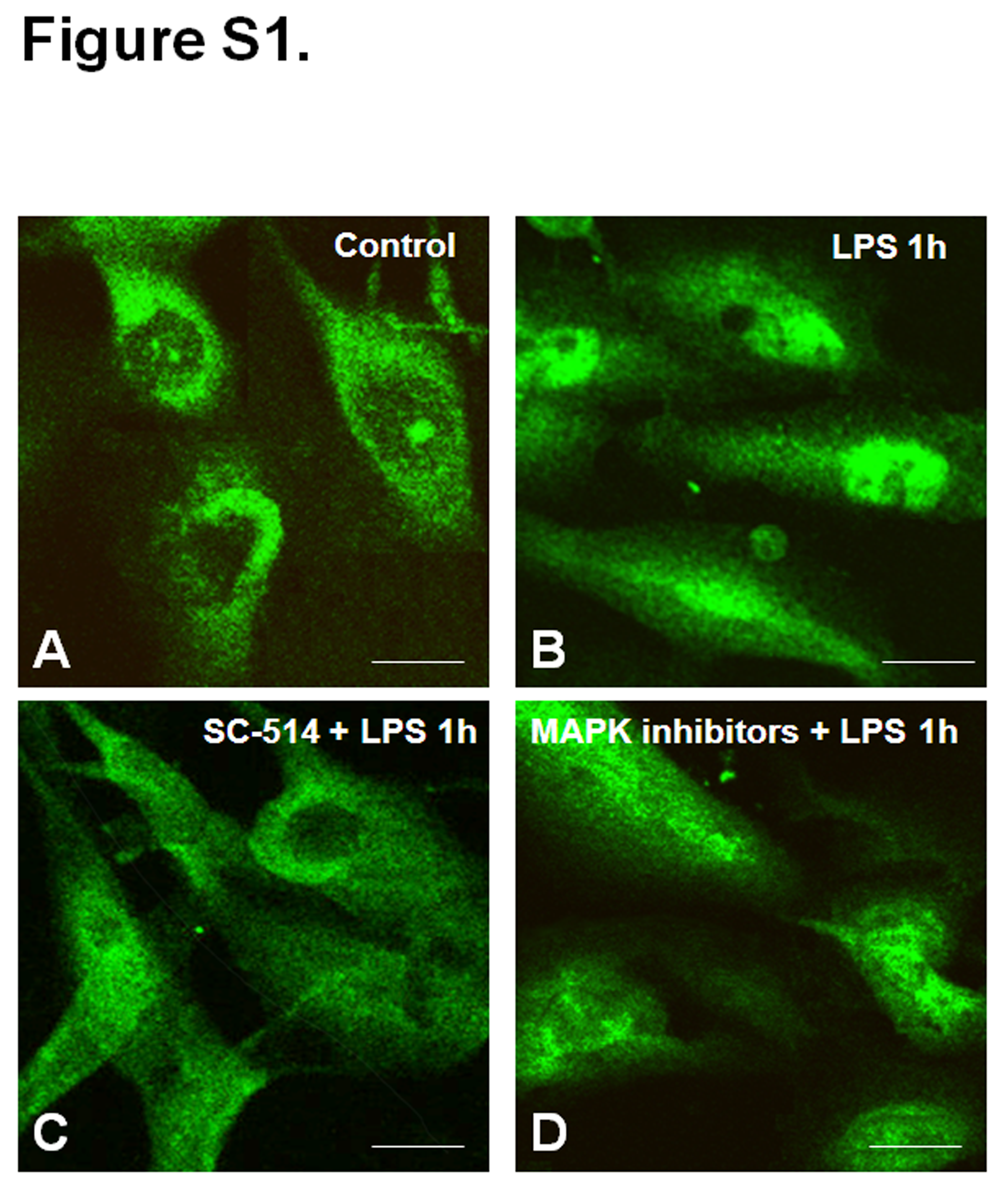

Supplement: Figure S1 — Effects of inhibitors to NF-κB and MAPK signaling pathways on LPS-induced nuclear translocation. H69 cells were treated with LPS for 1 h in the presence or absence of inhibitors, followed by immunofluorescent microscopy. p65 was stained in green. Bars = 5 µm. (TIF) [file pone.0030772.s001.tif]

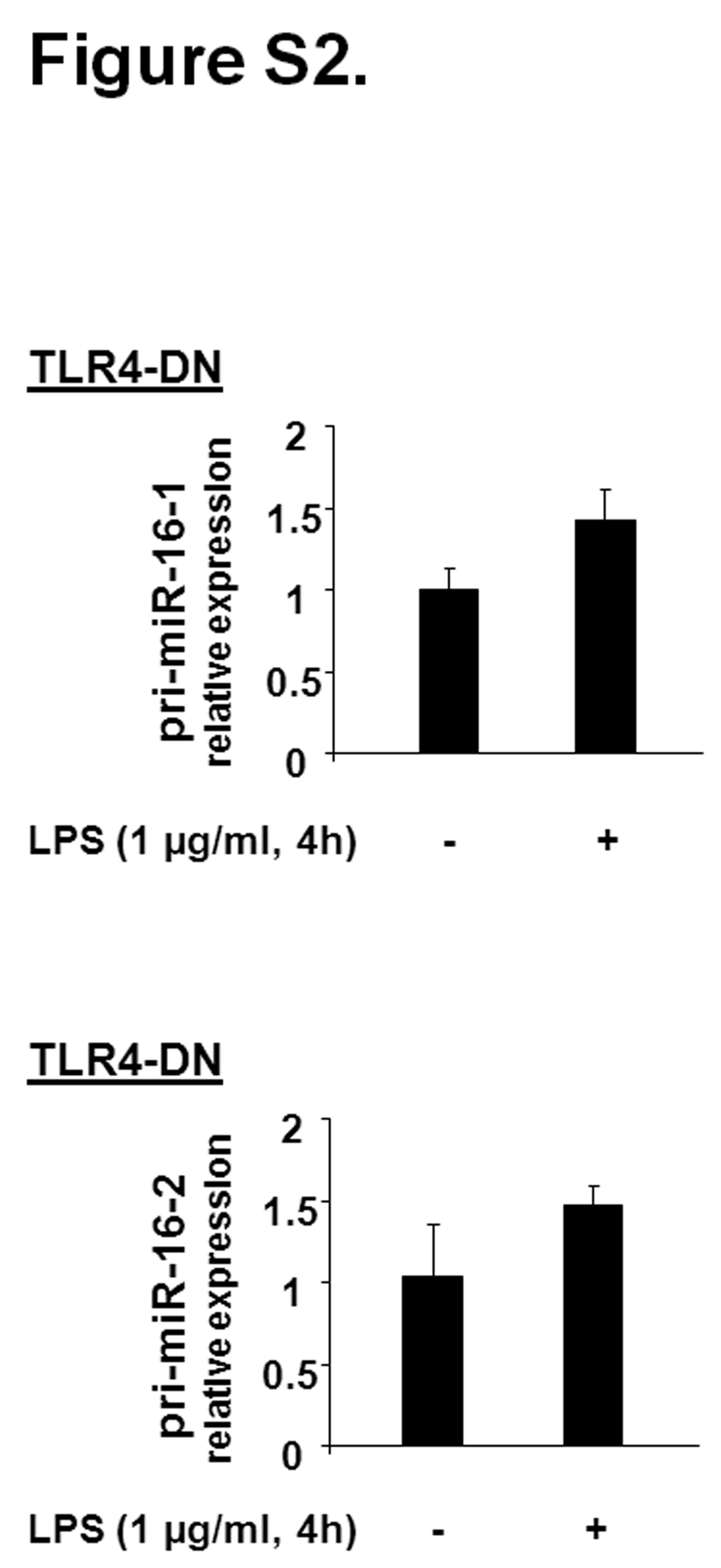

Supplement: Figure S2 — LPS stimulation does not increase expression of pri-miR-16-1 and pri-miR-16-2 in TLR4-DN H69 cells. The expression of pri-miR-16-1 and pri-miR-16-2 was measured by real-time PCR in H69 cells stably expressing TLR4-DN following LPS stimulation (1 µg/ml) for 4 h. Data shown are averages of three independent experiments. (TIF) [file pone.0030772.s002.tif]

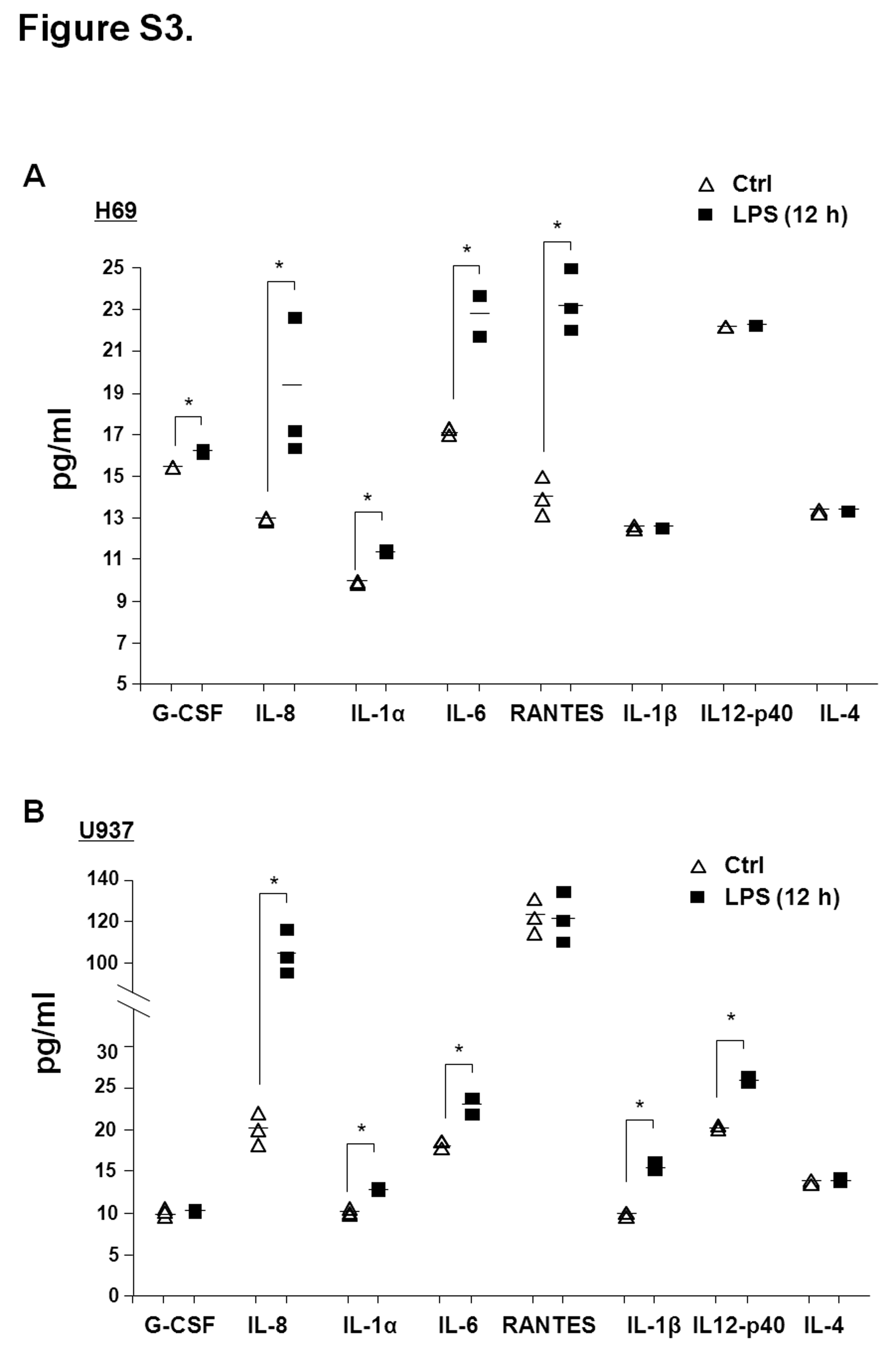

Supplement: Figure S3 — LPS stimulation increases expression of inflammatory cytokines and chemokines in H69 and U937 cells. Multiplex bead array analysis of inflammatory cytokines and chemokines in H69 (A) and U937 (B) cells following LPS stimulation (1 µg/ml) for 12 h. Data shown are averages of three independent experiments. ∗, p<0.05 t-test vs. non-stimulated cells. (TIF) [file pone.0030772.s003.tif]

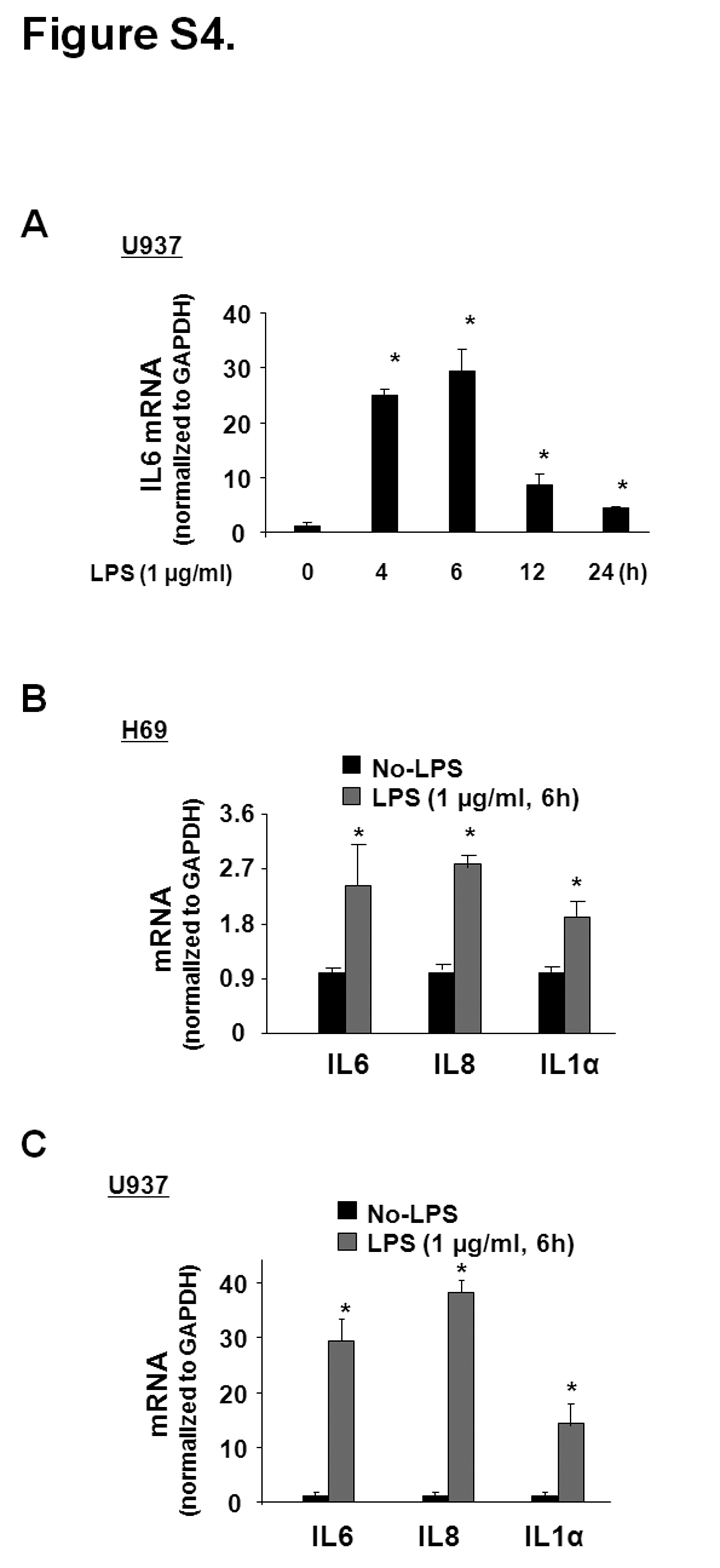

Supplement: Figure S4 — LPS stimulation increases mRNA levels of IL-6, IL-8, and IL-1α in H69 and U937 cells. (A) Time-dependent increase of IL-8 mRNA levels in U937 cells in response to LPS stimulation. (B) and (C) mRNA levels of IL-8, IL-6, and IL-1á were measured by real-time PCR in H69 and U937 cells following LPS stimulation (1 µg/ml) for 6 h. Data shown are averages of three independent experiments. ∗, p<0.05 t-test vs. the non-stimulated cells. (TIF) [file pone.0030772.s004.tif]

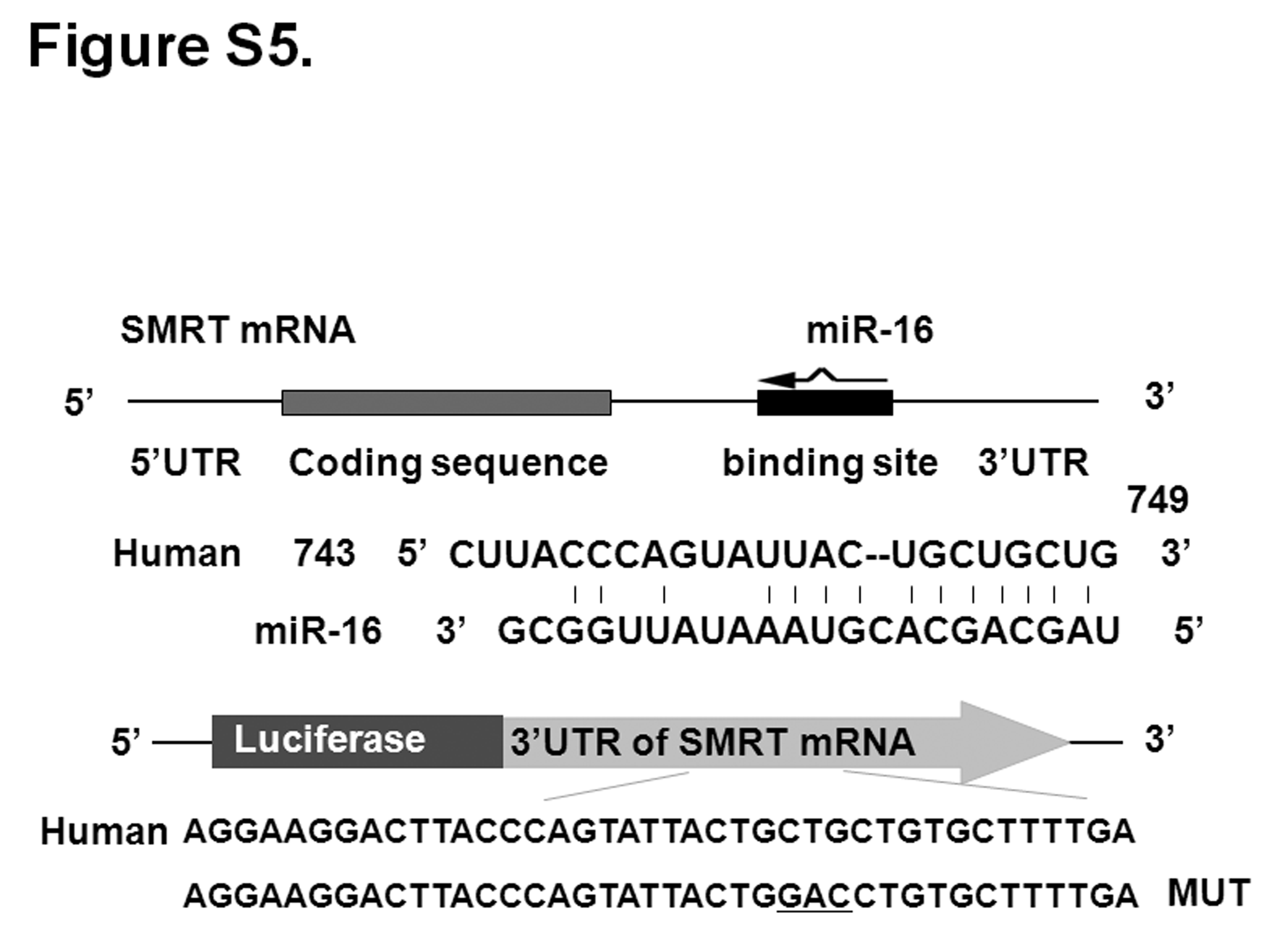

Supplement: Figure S5 — The schematic of SMRT mRNA indicates a potential binding site for miR-16 in the 3′UTR. The SMRT 3′UTR sequence encoding the potential miR-16 binding site was inserted into the pMIR-REPORT luciferase plasmid. A control plasmid with a mutant 3′UTR sequence was used as a control. (TIF) [file pone.0030772.s005.tif]

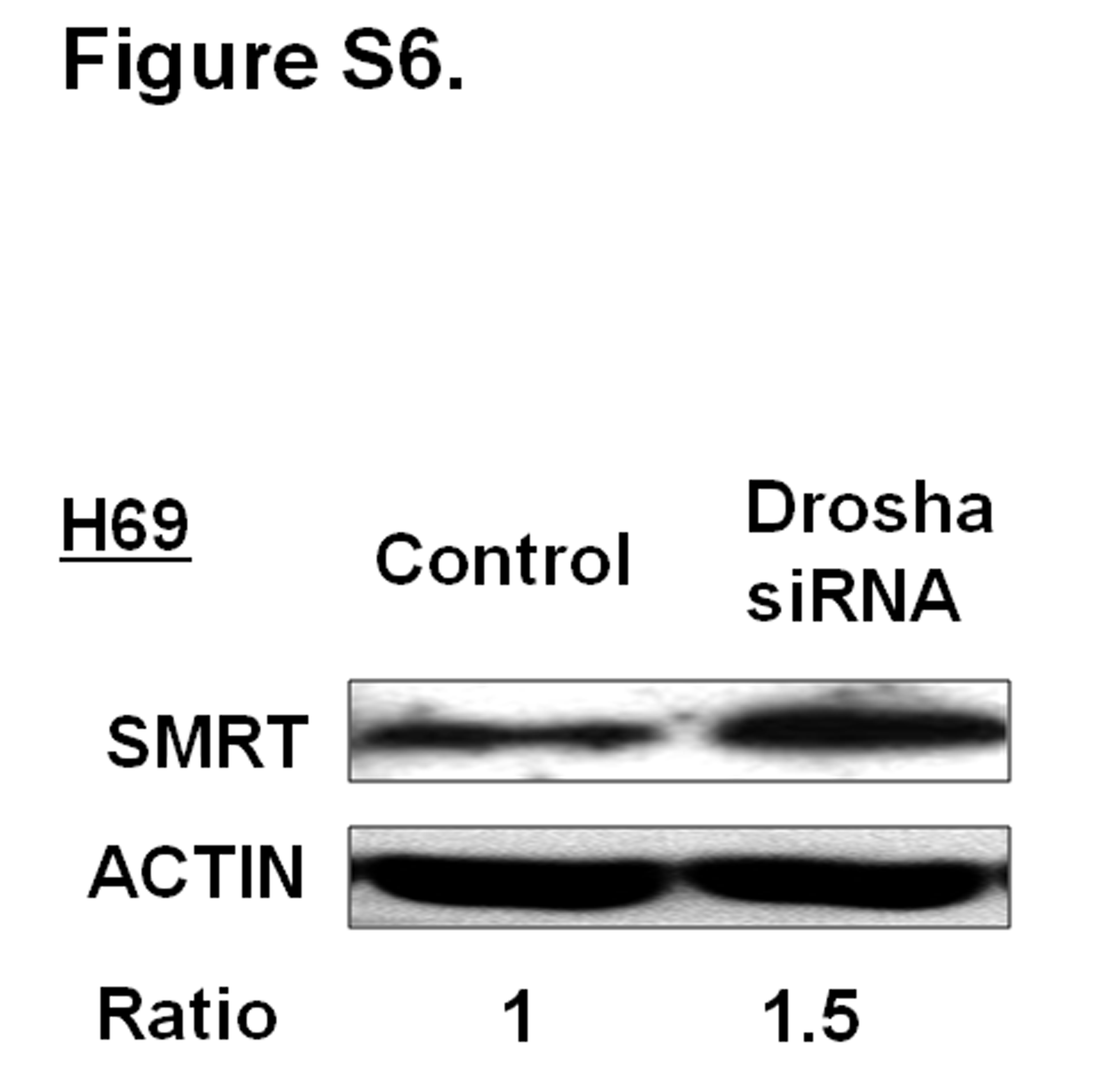

Supplement: Figure S6 — Drosha silencing induces the upregulation of SMRT expression at the protein level. H69 cells were transfected with a scramble control siRNA or Drosha siRNA for 48 h, followed by Western blot for SMRT. A representative Western blot from three independent experiments is shown. β-actin was also blotted to ensure equal loading. Densitometric levels of SMRT signals were quantified and expressed as their ratio to β-actin. (TIF) [file pone.0030772.s006.tif]

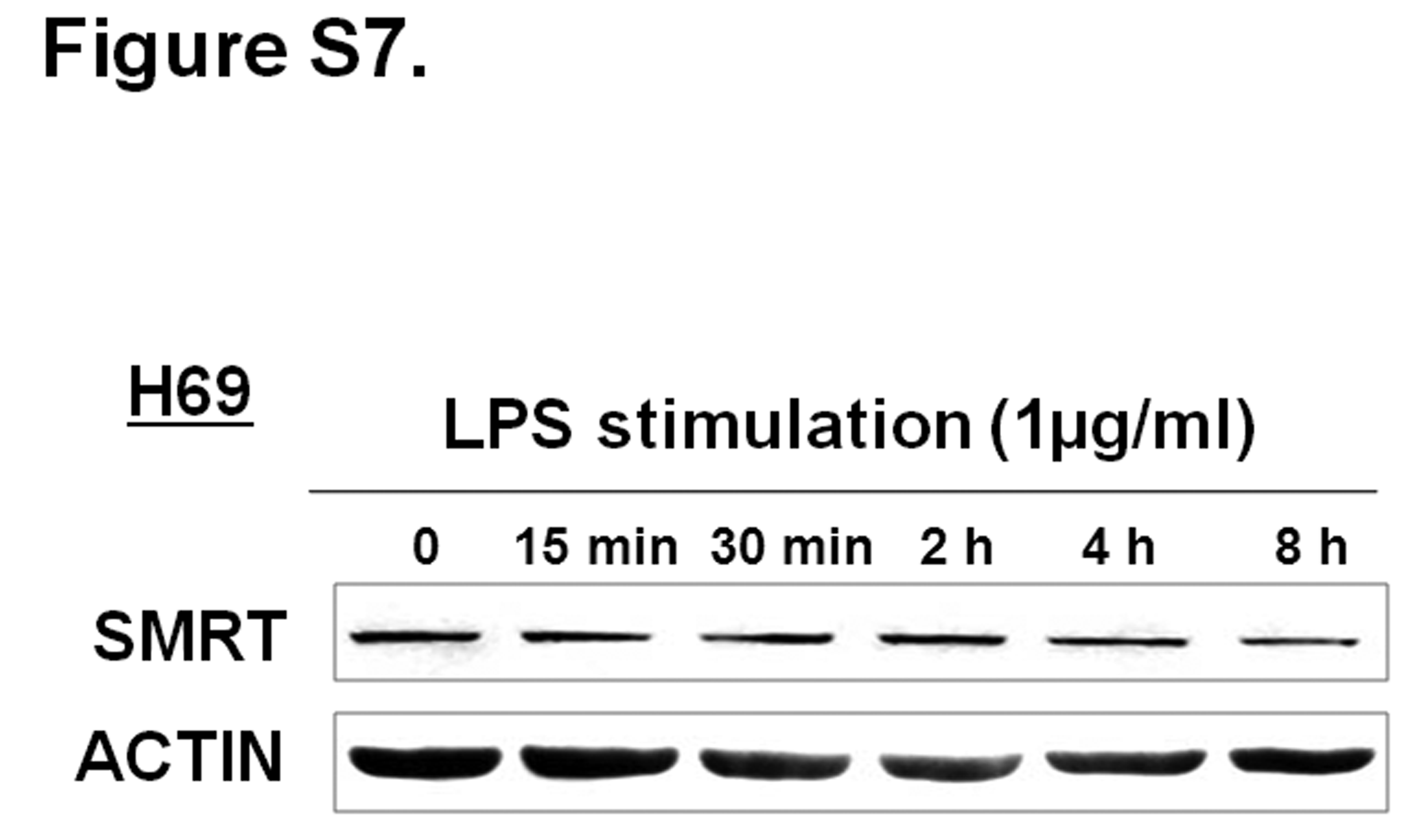

Supplement: Figure S7 — LPS stimulation does not decrease SMRT expression at early time points after LPS treatment. H69 cells were exposed to LPS for up to 8 h, followed by Western blotting for the SMRT protein. Representative blots from at least three independent experiments are shown. β-actin was blotted as a loading control. (TIF) [file pone.0030772.s007.tif]

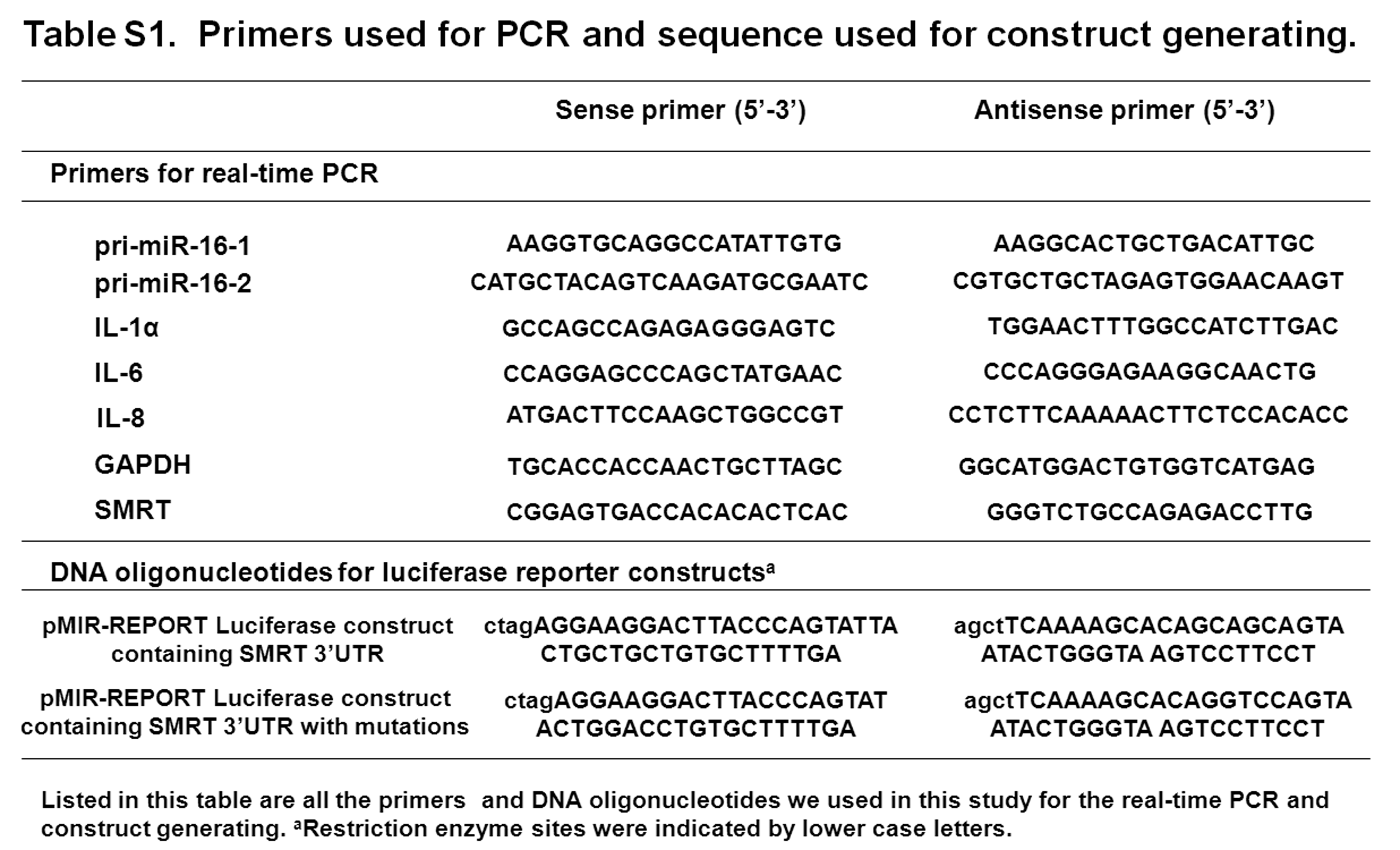

Supplement: Table S1 — Primers used for PCR and sequence used for construct generating. (TIF) [file pone.0030772.s008.tif]
